# Identification, Expression and Activity of Candidate Nitrite Reductases From Orange *Beggiatoaceae*, Guaymas Basin

**DOI:** 10.3389/fmicb.2019.00644

**Published:** 2019-03-29

**Authors:** Andrew Buckley, Barbara MacGregor, Andreas Teske

**Affiliations:** ^1^Department of Marine Sciences, University of North Carolina at Chapel Hill, Chapel Hill, NC, United States; ^2^Department of Earth Sciences, College of Science and Engineering, University of Minnesota, Minneapolis, MN, United States

**Keywords:** *Beggiatoaceae*, nitrite reductase, tetrathionate reductase, cytochrome, Guaymas Basin

## Abstract

Orange filamentous *Beggiatoaceae* form massive microbial mats on hydrothermal sediments in Guaymas Basin; these bacteria are considered to oxidize sulfide with nitrate and nitrite as electron acceptors. From a previously analyzed genome of an orange *Beggiatoaceae* filament, three candidate genes for enzymes with nitrite-reducing function – an orange octaheme cytochrome, a *nirS* nitrite reductase, and a nitrite/tetrathionate-reducing octaheme cytochrome – were cloned and expressed in *Escherichia coli*. The expressed and purified orange cytochrome showed reduced nitrite-reducing activity compared to the multifunctional native protein obtained from microbial mats. The *nirS* gene product showed *in vitro* but no in-gel nitrite-reducing activity; and the nitrite/tetrathionate-reducing octaheme cytochrome was capable of reducing both nitrite and tetrathionate *in vitro*. Phylogenetic analysis shows that the orange *Beggiatoaceae nirS*, in contrast to the other candidate nitrite reductases, does not form monophyletic lineages with its counterparts in other large sulfur-oxidizing bacteria, and most likely represents a recent acquisition by lateral gene transfer. The nitrite/tetrathionate-reducing enzyme of the orange *Beggiatoaceae* is related to nitrite- and tetrathionate reductases harbored predominantly by Gammaproteobacteria, including obligate endosymbionts of hydrothermal vent tubeworms. Thus, the orange Guaymas Basin *Beggiatoaceae* have a repertoire of at least three different functional enzymes for nitrite reduction. By demonstrating the unusual diversity of enzymes with a potential role in nitrite reduction, we show that bacteria in highly dynamic, sulfide-rich hydrothermal vent habitats adapt to these conditions that usually prohibit nitrate and nitrite reduction. In the case of the orange Guaymas *Beggiatoaceae*, classical denitrification appears to be replaced by different multifunctional enzymes for nitrite and tetrathionate reduction; the resulting ecophysiological flexibility provides a new key to the dominance of these *Beggiatoaceae* in hydrothermal hot spots.

## Introduction

Extensive microbial mats dominated by large filamentous sulfur-oxidizing *Beggiatoaceae* (Jannasch et al., 1989; Nelson et al., 1989) are among the most conspicuous features of hydrothermally active seafloor sediments and mounds at the Guaymas Basin spreading center in the central Gulf of California. The consistent oxygen depletion throughout the deep-water column and in the bottom water of Guaymas Basin (Calvert, 1964; Gundersen et al., 1992), coinciding with high nitrate/nitrite concentrations (Teske et al., 2016), create a highly suitable habitat for microaerophilic, nitrate- and nitrite-reducing microbial populations. Hydrothermal circulation mixes microoxic bottom water with sulfidic and low-molecular weight organic-rich hydrothermal fluid at this dynamic interface (Gundersen et al., 1992; Teske et al., 2016). These conditions enable the Guaymas Basin *Beggiatoaceae* to grow as centimeter-thick microbial mats on hydrothermal hot spots (Jannasch et al., 1989), in contrast to the millimeter-size interface habitat of most aerobic, sulfur-oxidizing bacteria (Jørgensen and Postgate, 1982).

Frequently, these bacterial mats show a distinct zonation on the seafloor that is reminiscent of fried eggs: orange-colored filaments of ca. 35–40 μm diameter in the central mat area are surrounded by a wide margin dominated by non-pigmented filaments reaching 100–120 μm diameter. These types are genetically and presumably also physiologically distinct, as their zonation appears to reflect different preferences for steep thermal and geochemical gradients in the center of a hydrothermal hot spot, versus more moderate gradients on the periphery (McKay et al., 2012). These particular *Beggiatoaceae* populations with consistent 16S rRNA gene sequences, color zonation and filament diameters have been observed consistently in every Guaymas Basin cruise by the author’s lab, in 1998, 2008, 2009 (McKay et al., 2012; Teske et al., 2016) and again in December 2016 and November 2018.

By 16S rRNA gene phylogeny, the Guaymas *Beggiatoaceae* form a well-supported cluster with other large, conspicuous marine sulfur-oxidizing bacteria, such as the genera *Thiomargarita* and *Marithioploca;* they are phylogenetically and physiologically distinct from the genus *Beggiatoa* in the strict sense, as represented by the type species, the heterotrophic freshwater species *Beggiatoa alba* (Teske and Salman, 2014). A common feature of large sulfur-oxidizing bacteria in the *Beggiatoaceae* is the possession of a cytoplasmic vacuole that fills almost the entire cell interior and limits the cytoplasm to a thin layer on the cell membrane; with few exceptions (Kalanetra et al., 2004), this vacuole serves as the receptacle for high (>100 mM) intracellular concentrations of nitrate; without this large vacuole, nitrate accumulation is absent (McHatton et al., 1996). Intracellular nitrate accumulation, combined with conspicuous, membrane-bound sulfur globules embedded in the cytoplasm, and a habitat preference for the surface of sulfide-rich sediments and nitrate-rich bottom water (Teske et al., 2016), supported the working theory that these bacteria are nitrate-reducing sulfide oxidizers that oxidize sulfide first to sulfur and then to sulfate; the previously studied model organism *Thioploca* (revised to Marithioploca, Salman et al., 2013) reduced nitrate to ammonia (Otte et al., 1999).

Genome sequencing of the orange Guaymas *Beggiatoaceae* resulted in a non-closed genome of ca. 4.5 million bases, encoding pathways for sulfide oxidation, nitrate respiration, inorganic carbon fixation by both Type II RuBisCO and the reductive tricarboxylic acid cycle, acetate and possibly formate uptake, and energy-generating electron transport via both oxidative phosphorylation and the Rnf complex (MacGregor et al., 2013a). An orange-colored dominant protein isolated from orange Guaymas Basin *Beggiatoaceae* mats, an octaheme cytochrome oxidase, matched an ORF on this genome (ORF 00024_0691), and showed strong homologies to octaheme cytochrome genes in different gammaproteobacterial sulfur-oxidizing bacteria, purple sulfur bacteria, and *Shewanella* (MacGregor et al., 2013b). The gene also shared heme binding sites and unusual active sites marked by lysine residues with hydroxylamine and hydrazine oxidases, the key enzymes of aerobic and anaerobic ammonia oxidation; the native orange protein turned out to have these activities but in addition showed nitrite-reducing activity (MacGregor et al., 2013b).

The genomic context of the orange *Beggiatoaceae* showed no evidence for ammonia oxidation pathways, as in aerobic nitrifying bacteria. However, seven candidate ORFs for membrane-bound and periplasmatic nitrate reductases were found (MacGregor et al., 2013a), indicating that nitrite can be generated from nitrate, and is subsequently available for nitrite reduction. Candidate predicted proteins for the nitrite reduction pathway include a potential nirS-type nitrite reductase (ORF 00500_2967) with weak homology to nirK, and candidate ORFs for nitric oxide reductases (norB and norC) leading to nitrous oxide as the end product. An alternate pathway is suggested by the presence of a periplasmatic octaheme cytochrome c reductase (ORF 01341_2386) with potential nitrite and hydroxylamine reductase activity producing ammonia (MacGregor et al., 2013a).

In this study, we phylogenetically characterize, and express these three candidate genes coding for potential nitrite reductases obtained from orange Guaymas Basin *Beggiatoaceae (ORFs BOGUAY_2386, 2967, and* 0691), and compare the activity and properties of these candidate enzymes with the native nitrite-reducing orange octaheme cytochrome that has been obtained and purified previously from the same organism (MacGregor et al., 2013b). Investigating the nitrite reduction potential of these potential nitrite reductases is providing new insights into the ecophysiology of Guaymas Basin *Beggiatoaceae* linking the benthic carbon, sulfur and nitrogen cycles in this hydrothermal habitat.

## Materials and Methods

### Habitat Characteristics and Sampling Site

**FIGURE 1:**
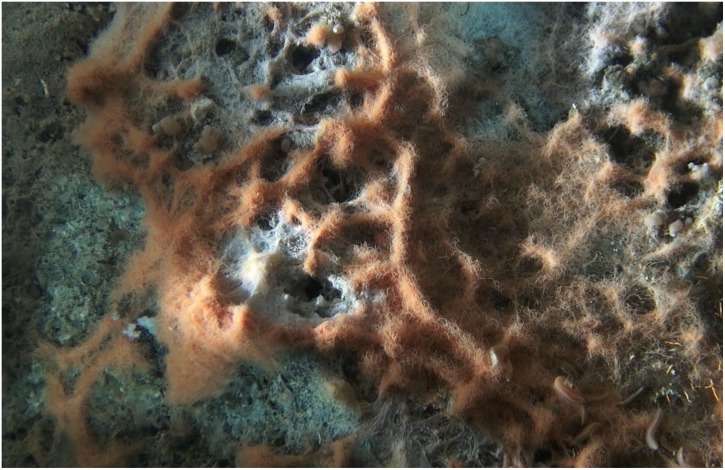
*In situ* close-up video still photo of an orange *Beggiatoaceae* mat in Guaymas Basin, taken by the bottom-facing *Alvin* camera a few centimeters above the seafloor during *Alvin* dive 4872 in the Cathedral Hill area of Guaymas Basin (27°N00.70/111°W24.25). The image shows the complex three-dimensional structure of a mat, arranged around several small hydrothermal outflow areas marked by white sulfur deposits, visible in the center of the image. The Individual orange filaments have approx. 40 μm diameter.

The Guaymas *Beggiatoaceae* colonize sediment surface interfaces that provide nitrate and nitrite in concentrations of ca. 20–65 μM *in situ*, mM concentrations of ammonia in the underlying sediment, and oxygen in low concentrations of maximally 30–60 μM, corresponding ca. 10–20% of seawater saturation, in the overlying water column (Figure 1 and Table 1). An orange *Beggiatoaceae* tuft was retrieved from hydrothermal sediment core collected on Alvin Dive 4568 during RV *Atlantis*/HOV *Alvin* cruise AT15-56 on November 19, 2009 in Guaymas Basin, Gulf of California, Mexico (latitude 27°00.444300N, longitude 111°24.542700W; depth, 2002 m). One of the filaments was purified of attached bacteria, and its genome was amplified and sequenced as described (MacGregor et al., 2013a,b).

**Table 1:** Nitrate, nitrate/nitrite and ammonia concentrations for the sediment/water interface and *Beggiatoaceae* mats in Guaymas Basin.

	*In situ* oxygen [μM]	*In situ* nitrate + nitrite [μM]	*Ex situ* nitrate [μM]	Ammonia [μM]	References
Bottom water	30–60^∗^	20^∗^	Water samples, 6–54^∗∗^	Water samples, 30–80^∗∗^	Teske et al. (2016)^∗^, Winkel et al. (2014)^∗∗^, McKay et al. (2012)^∗∗∗^, and Schutte et al. (2018)^∗∗∗∗^
*Beggiatoaceae* mat	Locally variable, 0–10^∗,∗∗^	≤ 65 ^∗,∗∗^	Intracellular, ca. 50000^∗∗∗^ Porewater, ca. 500–1500^∗∗∗∗^	—	
Hydrothermal Sediment under mat	none^∗∗^		Porewater in top 5 cm, 20–48^∗∗^ Declining to 1–5 at 25 cm^∗∗∗∗^	Porewater in top 5 cm, 50–5300^∗∗^; downcore at 25 cm, 1300–4200 ^∗∗∗∗^	


*“In situ” designates data from in situ microprofiler analysis. “intracellular” nitrate concentrations come from the analysis of single, vacuolated filaments; “Porewater” designates sediment porewater, and “water samples” indicate Niskin bottle samples taken on the seafloor of Guaymas Basin. Data are referenced with one, two, three or four asterisks, indicating Teske et al. (2016), Winkel et al. (2014), McKay et al. (2012), and Schutte et al. (2018), respectively.*

### Target Genes

Annotated genome sequences were originally referred to by 5-digit contig number and 4-digit open reading frame (ORF) number, e.g., 00024_0691; the genome was called the BOGUAY genome based on the IMG/ER acronym, derived from “*Beggiatoa* orange Guaymas.” Here, we use the 4-digit open reading frame number together with the BOGUAY acronym, for consistency with previously published analyses (MacGregor et al., 2013a,b). Three candidate nitrite reductase genes were investigated: the orange transmembrane multiheme cytochrome BOGUAY_0691 (00024_0961), the candidate nitrite reductase NirS BOGUAY_2967 (00500_2967) (MacGregor et al., 2013b), and a potentially multifunctional tetrathionate/nitrite reductase BOGUAY_2386 (01341_2386) (Mowat et al., 2004; Tikhonova et al., 2006).

### PCR Amplification and Cloning Strategy

The BOGUAY_2967, BOGUAY_2386, and BOGUAY_0691 target genes including their flanking regions were PCR-amplified with primer combinations 2967 EXP F and R, 2386 EXP F and R, and 0691 EXPF and R (primer pairs 1–3, Table 2) from genomic amplified DNA of a single orange filament, collected during Alvin dive 4568; initial PCR amplifications with primers that excluded the flanking regions were not successful (primer pairs 4–6, Table 2). The “flanking region” PCR products were then cloned into a pCR-XL-TOPO vector (Thermo Fisher) and again PCR-amplified, now with primers 2967 F and R, 2386 F and R, and 0691 F and R (primer pairs 4–6, Table 2) located in the terminal regions of the target genes and excluding the flanking regions. These primers resulted in shorter but consistently retrieved PCR amplicons. The resulting PCR products were cloned into vector plasmid pCR2.1 (Supplementary Table 1), transformed into *Escherichia coli* and grown to obtain inserts without flanking regions. For subsequent gene expression, the target genes (now without flanking regions) were put into the pET22b vectors for expression in *E. coli* strain BL21. The primer pairs 4–6 were modified by adding different restriction sites to forward and reverse versions (primer pairs 7–9), for cloning the target genes into pET22b with correct directionality.

**Table 2:** PCR Primers, PCR targets, PCR annealing temperatures, and amplicon lengths (including primer sequences) for candidate nitrite reductase genes.

Primer Name and pair	Primer Sequence 5′→3′	Annealing temperature	Primer target Application	amplicon length
2967 EXP F (pair 1)	CAACAAATGTGAAATAAAGAG	53°C	BOGUAY_2967 and flanking regions	2483 bp
2967 EXP R (pair 1)	TCTAAAAATAAATGTGGTCTG	53°C	BOGUAY_2967 and flanking regions	2483 bp
2386 EXP F (pair 2)	TCCACCTTAACTAACTCACT	57°C	BOGUAY_2386 and flanking regions	1878 bp
2386 EXP R (pair 2)	GAATAAATATCAGAGCCGCT	57°C	BOGUAY_2386 and flanking regions	1878 bp
0691 EXP F (pair 3)	AAAAATGGTTGGCAGGTGGG	57°C	BOGUAY_0691 and flanking regions	1797 bp
0691 EXP R (pair 3)	ACTTTGCACCCCACATTACC	57°C	BOGUAY_0691 and flanking regions	1797 bp
2967F (pair 4)	ATGAGATATATTAGTCATTTCC	52°C	BOGUAY_2967	2016 bp
2967R (pair 4)	TTAATAAATGTCATGCGTCG	52°C	BOGUAY_2967	2016 bp
2386F (pair 5)	ATGATAAAAAAACAGCTCGC	52°C	BOGUAY_2386	1748 bp
2386R (pair 5)	TTTTTCACCTCCAATATGACAG	52°C	BOGUAY_2386	1748 bp
0691F (pair 6)	ATGATTCGTAAATTGTGGGC	54°C	BOGUAY_0691	1536 bp
0691R (pair 6)	TCTATCGGTTTGCTCACCATA	54°C	BOGUAY_0691	1536 bp
2967 FRE (pair 7)	CCCGTTCCATGGATGAGATATATTAGTCATTTCC	59°C	NcoI restriction site on BOGUAY_2967	2046 bp
2967 RRE (pair 7)	AAAGGGCCATGGCTCGAGATAAATGTCATGCGTCG	59°C	XhoI restriction site on BOGUAY_2967	2046 bp
2386 FRE (pair 8)	CCCGGGCCATGGATGATAAAAAAACAGCTCGC	63°C	NcoI restriction site on BOGUAY_2386	1778 bp
2386 RRE (pair 8)	AATAGTCCATGGCTCGAGTTTTTCACCTCCAATATGACAG	63°C	XhoI restriction site on BOGUAY_2386	1778 bp
0691 FRE (pair 9)	AAGCTTATGATTCGTAAATTGTGGGC	63°C	HindIII restriction site on BOGUAY_0691	1548 bp
0691 RRE (pair 9)	CTCGAGTCTATCGGTTTGCTCACCATA	63°C	XhoI restriction site on BOGUAY_0691	1548 bp
T7prom	TAATACGACTCACTATAGGG	60°C	pET22b Promoter region	


### PCR Conditions

PCR mixtures contained 1 μL of 1:10 diluted DNA extract, 2 μL of each primer (10 pmol/μL), 2 μL of deoxynucleotide triphosphates (dNTPs) (10 mM each), 5 μL of 10 × PCR buffer (final concentration 1.5 mM MgCl2), 2 μL of bovine serum albumin (BSA, 10 mg/mL), 0.15 μL of Taq polymerase (5 U/μL) (Promega, Madison, WI, United States) and sterile water to a final volume of 50 μL. The PCR amplification was performed using an iCycler (Bio-Rad, Hercules, CA, United States). The PCR mix was incubated at 94°C for 4 min, followed by 35 cycles of denaturation at 94°C for 1 min, annealing at different temperatures (Table 1) for 1 min, and extension at 72°C for 1 min. PCR amplification ended with a single 10 min extension step at 72°C. The PCR products were evaluated by gel electrophoresis on 1.5% agarose gels stained with ethidium bromide (0.5 mg/L).

### Cloning of PCR-Amplified Genes

The PCR products were gel purified with the Promega Wizard SV Gel Clean-Up System (Promega Corp., Madison, WI, United States) following the manufacturer’s instructions in order to remove contaminants in the sample that could have interfered with cloning. The purified PCR products were ligated into TOPO XL Cloning Vector plasmids containing β-galactosidase and kanamycin resistance genes. *E. coli* strains were made electrocompetent as described previously (Gonzalez et al., 2013) and subsequently transformed with their respective plasmids using a Micropulsor (Bio-Rad, Berkeley, CA). Transformed strains were incubated in SOB medium and plated onto Luria Broth (LB) medium agar plates containing bromo-chloro-indolyl-galactopyranoside (X-GAL) and kanamycin for blue/white screening. After 24 h of incubation at 37°C, white colonies were picked. The colonies were re-plated after another 24 h for control PCR reactions using the same PCR primers as before. As a precaution, copies of the colonies with the desired inserts were placed in glycerol stocks (85% S.O.C. medium and 15% glycerol) and stored at –80°C for resequencing if required. Plasmids containing the desired inserts were isolated using GeneJET Plasmid Miniprep Kit (Thermo Scientific, Waltham, MA). The plasmid inserts were verified for correct sequence and direction (Genewiz, South Plainfield, NJ). PCR product inserts, and all plasmids used in this study, are listed in Supplementary Table 1. A complete list of strains used in this study is given in Supplementary Table 2.

### Molecular Biology Software Tools

DNA oligonucleotides were developed with MacVector (Apex, NC). Restriction site modifications were chosen by the results of NEB Cutter v2.0^1^. SignalP was used to predict the extent of signal peptides vs. transmembrane protein domains^2^ (Petersen et al., 2011). Protein threading was performed by the online available MUSTER program (Wu and Zhang, 2008). Protein sequences were trimmed of the predicted signal sequence predicted using SignalP, prior to MUSTER submission. Protein sequences were submitted to MUSTER to align and score the most likely protein-folding model. The highest *z*-score protein threading alignment was chosen and visualized using free-source molecular graphics software iMol^3^.

### Sequence Alignments

Translated protein alignments were made with MEGA using the MUSCLE algorithm (Kumar et al., 2016), then edited to align possible heme binding domains. Alignment colors for amino acids were generated using the Jalview program (Waterhouse et al., 2009) and the Clustal color option. Homologs of the BOGUAY sequences were identified by BLASTP searches (Version 2.7) and by blastX DNA vs. Protein searches (Version 5.570, March 2017) of the IMG/ER^4^ and NCBI databases. The nucleotide equivalents of these protein alignments were used for phylogenetic tree inference. The full sequence alignments are provided as Supplementary Figures 1–3, annotated with gene numbers for genome-derived sequences as archived in IMG/ER^5^.

### Phylogenetic Analysis

Protein-based phylogenetic trees were inferred by using the Maximum Likelihood method based on evolutionary distances computed using the Poisson correction method (Zuckerkandl and Pauling, 1965). Initial trees for the heuristic search were obtained automatically by applying Neighbor-Join and BIONJ algorithms to a matrix of pairwise distances estimated using a JTT model, and then selecting the tree topology with superior log likelihood value. The percentage of replicate trees in which the associated taxa clustered together were determined by bootstrap testing with 1000 replicates (Felsenstein, 1985). Protein-based evolutionary distances are given in the units of the number of amino acid substitutions per site. All positions with less than 95% site coverage were eliminated. All analyses were performed in the program package MEGA7 (Kumar et al., 2016).

### Protein Expression and Purification

Recombinant proteins were expressed and isolated from their respective *E. coli* strains as previously described (Hendriksen et al., 2008; Villapakkam et al., 2009; Santiago et al., 2013). Briefly, the *E. coli* expression strains BL210691OGB, BL212386OGB, and BL212967OGB were grown to exponential phase (OD_600_ ∼0.6) in SOB medium supplemented with ampicillin to a final concentration of 100 μg/mL. Isopropyl ß-D-thiogalactopyranoside (IPTG) (EMD Millipore, Billerica, MA) was added to the culture to a final concentration of 1 mM, and cells were grown for 8 h at 30°C.

Following expression, the BOGUAY_0691 gene product – the multifunctional soluble orange protein (MacGregor et al., 2013b) – and the BOGUAY_2386 gene product – the octaheme cytochrome C reductase protein (termed ONR gene product in MacGregor et al., 2013a) – were isolated from the BL210691OGB and BL212386OGB expression strains using previously described protocols under aerobic conditions (Hendriksen et al., 2008; Villapakkam et al., 2009; Santiago et al., 2013). Briefly, the procedure involved lysozyme addition, three freeze-thaw cycles, and ten sonication cycles of 20 s each. For purification of the BOGUAY_2386 gene product, the non-ionic detergent Triton-X was added after the lysozyme step but before the freeze-thaw steps, to envelop and solubilize hydrophobic protein domains. Expression of the BOGUAY_2967 gene product in expression strain BL212967OGB was induced in the same way as BOGUAY_2386 and BOGUAY_0691. However, efforts to purify and isolate the enzymatically active BOGUAY_2967 gene product – the nirS candidate protein (MacGregor et al., 2013a) – in the same non-denaturing manner as the other two gene products were unsuccessful. Purification was only achieved under denaturing and reducing conditions with urea and SDS, but non-ionic detergents did not work.

### In-Gel Enzymatic Staining

For in-gel activity assays, non-denaturing polyacrylamide gels were used; gels run were performed in an anaerobic glove box under N_2_. To measure peroxidase activity, the polyacrylamide gels with freshly run protein samples were submerged in sodium-acetate buffer pH 6.0, and bubbled with nitrogen gas for 10 min. 50 mg of 3,3′-diaminobenzidine (DAB) was dissolved in the solution. To start the reaction, 900 μL of 30% H_2_O_2_ was added to the solution and the color allowed to resolve from 4 h to overnight at 4°C. For the nitrite reductase assay, gels were processed similarly to the peroxidase assays, however, the buffer contained 0.1 M potassium phosphate pH 6.2, 10 mM sodium nitrite, and 0.3 mM methyl viologen (MV). The reaction was started with the addition of 1.0 mM sodium-dithionite as electron donor to reduce MV; sodium dithionite is a suitable reductant as it does not auto-reduce nitrite under enzymatically relevant conditions (Basu et al., 2008; Salhany, 2008).

### *In vitro* Spectrophotometric Measurements

All spectrophotometric assays were performed at room temperature in a positive pressure anaerobic chamber filled with nitrogen gas. Peroxidase activity was measured spectrophotometrically as previously described (Pütter and Becker, 1983; Keesey, 1987) using an UV/Vis spectrophotometer Genesys 10S UV-Vis (Thermo Scientific, Waltham, MA). The sample was added to a 1 mL reaction solution containing 100 mM potassium phosphate pH 5.0, 8.7 mM 2,2′-Azino-bis (3-Ethylbenzothiazoline-6-Sulfonic Acid) (ABTS), 3.2 mM hydrogen peroxide, 0.004% (w/v) bovine serum albumin, and 0.008% (v/v) Triton X-100. The reaction took place at 25°C over 120 s. Absorbance was monitored at 405 nm with an extinction coefficient of ABTS at 36.8 mM^-1^cm^-1^. Nitrite reductase activity was measured spectrophoto-metrically as previously described (Kostera et al., 2010; MacGregor et al., 2013b). The sample was added to a 1 mL reaction solution containing 100 mM potassium phosphate pH 7.0, 0.6 mM MV, 10.0 mM sodium nitrite, and 3.23 mM sodium dithionite. The reaction took place at 25°C over 120 s. Absorbance was monitored at 405 nm for the nitrite reducing conditions with an extinction coefficient for MV at 4.562 mM^-1^cm^-1^. Every measurement was performed with a blank control for abiotic, atmospheric oxidation of MV that contained no cell extract, and with an expression baseline control containing an extract of non-transformed *E. coli* cells; this expression baseline was always subtracted from measurements with induced *E. coli* cells, to take the biomass correction into account. Since cell lysates absorb at the same wavelength as MV and thus introduce a background absorption level that obscures low-level MV oxidation, only MV oxidation (destaining) curves above this threshold were used for activity determinations.

Tetrathionate reductase activity was measured spectrophotometrically as previously described (Hensel et al., 1999). The sample was added to a 1 mL reaction solution containing 10 mM potassium phosphate pH 7.4, 2 mM Na_2_EDTA, and 1.0 mM MV that had been titrated to A_600_∼1.5 with 100 mM sodium pyrophosphate pH 9.0, 50 mM sodium dithionite. The reaction was started with the addition of 500 μM potassium tetrathionate and monitored at 25°C for several minutes. Absorbance was monitored at 600 nm with an extinction coefficient for MV, under tetrathionate-reducing conditions, at 13 mM^-1^cm^-1^. Every measurement was performed with a blank control for abiotic, atmospheric oxidation of MV (sample added), as well as a control for direct MV oxidation by tetrathionate.

## Results

### Target Genes

The candidate nitrite reductase genes studied here included open reading frames BOGUAY_0691 (contig No. 00024_0961), matching a soluble orange transmembrane multiheme cytochrome with nitrite reductase, hydroxylamide oxidase and hydrazine oxidase activity (MacGregor et al., 2013b); BOGUAY_2967 (Contig No. 00500_2967) coding for a candidate nitrite reductase NirS, specifically periplasmic nitrite-reducing cytochrome *cd1* (MacGregor et al., 2013b); and BOGUAY_2386 (contig No. 01341_2386) coding for a octaheme cytochrome *c* tetrathionate/nitrite reductase of a type that was originally described as a tetrathionate reductase in *Shewanella oneidensis* (Mowat et al., 2004). In this soluble periplasmatic *Shewanella* enzyme, heme arrangements resembled those in ammonia-producing nitrite reductases and hydroxylamine oxidoreductases (Mowat et al., 2004), a prediction confirmed when *in vitro* tests demonstrated that nitrite and hydroxylamine were indeed reduced to ammonia (Atkinson et al., 2007; Einsle, 2011). A second example of this enzyme type was independently described for *Thioalkalivibrio nitratireducens* (Tikhonova et al., 2006).

**FIGURE 2:**
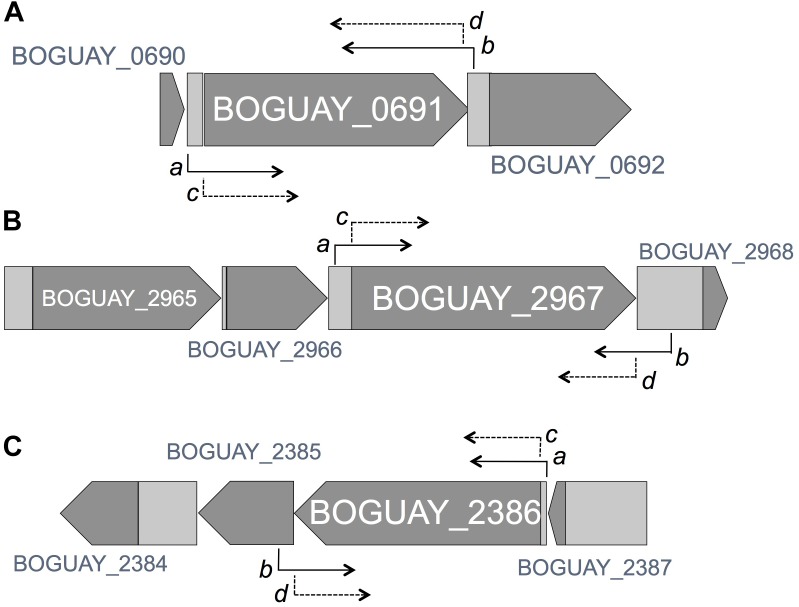
Genomic arrangement and context of target genes (ORFs BOGUAY_2967, 2386 and 0691), with arrows denoting the position of PCR primers pairs including and excluding flanking regions Genomic maps are derived from the GOLD Project ID# Gp0006298 on the IMG database. **(A)** A subset of the 41659 bp BOGUAY_Contig_00024 contains BOGUAY_0691 gene which translates into a 511aa protein. Primer 5′ ends are shown for pair 3, 0691 ExpF (*a*, 225 bp upstream of translational start site) and 0691 ExpR (*b*, ends 38 bp downstream of translational stop codon); and pair 6, 0691F (*c*, 5′-end at the start codon) and 0691R (*d;* 5′-end at the end of the gene, excluding the stop codon). **(B)** A subset of the 12025 bp BOGUAY_Contig_00500 contains the BOGUAY_2967 gene, which translates into a 671aa protein. Primer 5′ ends are shown for primer pair 1, *2967* ExpF (*a;* 184 bp upstream of translational start site) and 2967 ExpR (*b;* ends 281 bp downstream of translational stop codon); and for primer pair 4, 2967F (*c*, 5′-end at the start codon) and 2967 R (*d*; 5′-end at the end of the gene, excluding the stop codon). **(C)** A subset of the 15900 bp BOGUAY_Contig_01341 contains the BOGUAY_2386 gene, which translates into a 582aa protein. Primer 5′ ends are shown for pair *2*, 2386 ExpF (*a*, 40 bp upstream of translational start site), and 2386 ExpR (*b*, ends 91 bp downstream of translational stop codon); and for primer pair 5, 2386F (*c*, 5′-end at the start codon) and 2386R (d, position 5′-end at the end of the gene, excluding the stop codon).

The ORFs surrounding the target genes code for hypothetical proteins and enzymes with inferred functions outside of nitrite reduction pathways (Figure 2). Genes upstream of BOGUAY_0691 are predicted to encode periplasmatic nitrate reductase NapA and NapB subunits, a NADH dehydrogenase subunit, Fe-S and proton-translocating NADH-quinone oxidoreductases; downstream genes consist of conserved hypotheticals and functionally diverse genes (Supplementary Table 3). Genes upstream of BOGUAY_2967 include hypothetical proteins and a MO-CO oxidoreductase, downstream genes consist of viral insertions as well as hypotheticals; no genes were related to nitrate or nitrite reduction. Downstream of the BOGUAY_2386 tetrathionate reductase several genes with inferred functions in sulfur reduction were found, including a thiosulfate reductase cytochrome *b* subunit (ORF 2885; 68% similarity to *Sedimenticola* spp.), a thiosulfate reductase / polysulfide reductase chain A (ORF 2383, 75% similarity to *Beggiatoa* filament PS), a thiosulfate reductase subunit B (ORF 2382, 81% similarity to *Beggiatoa* filament PS) or Fe-S cluster containing dehydrogenase (80% and 73% similarity to 4Fe–4S ferredoxin in *Thiomargarita* and *Thiothrix*, respectively), and a Thiosulfate reductase subunit C (ORF 2381, 90% similarity to *Beggiatoa* filament PS; all % similarity values based on BlastP searches); upstream genes included hypotheticals, a sulfide-quinone oxidoreductase, and serine O-acetyltransferase (Supplementary Table 3).

### Phylogenetic Placement

**FIGURE 3:**
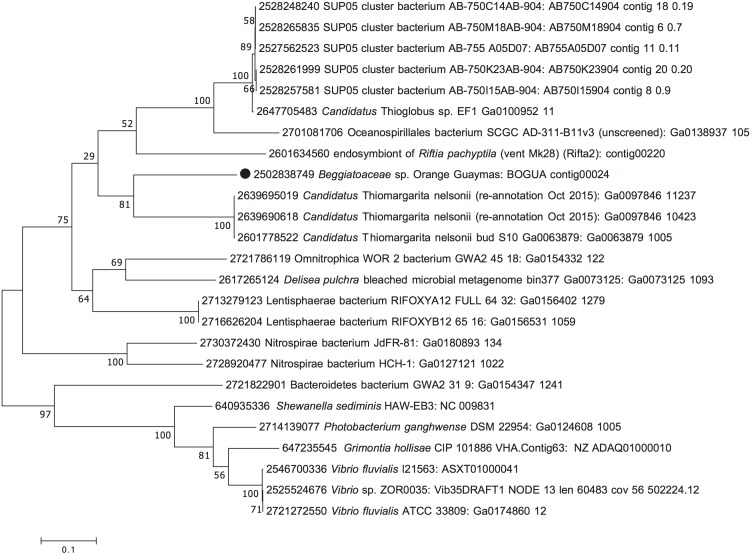
Maximum Likelihood phylogeny for the orange octaheme cytochrome (BOGUAY_0691), based on aligned protein sequences obtained by translating the gene. The scale bar shows the number of amino acid substitutions per site. Tree topology was tested with 1000 bootstrap replicates, and branching points are annotated with the percentages of recovering each node. Taxon labels start with IMG Gene ID numbers, followed by species, strain, or sequence/phylotype designations, and concluded with genomic ID and contig number as in IMG, unless omitted for sequence entries sharing the same origin.

**FIGURE 4:**
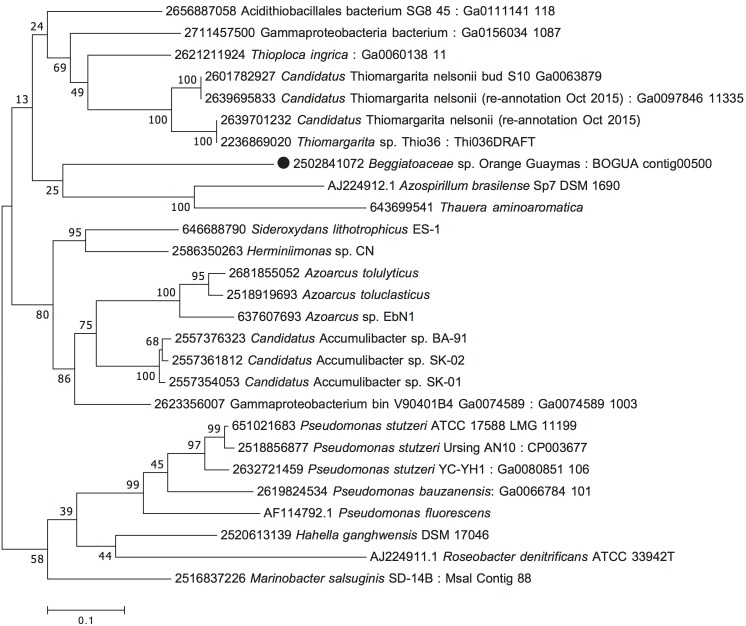
Maximum Likelihood phylogeny for candidate nitrite reductase NirS (BOGUAY_2967), based on aligned protein sequences obtained by translating the gene. Scale bar, bootstrap and taxon label annotation are the same as in Figure 3.

**FIGURE 5:**
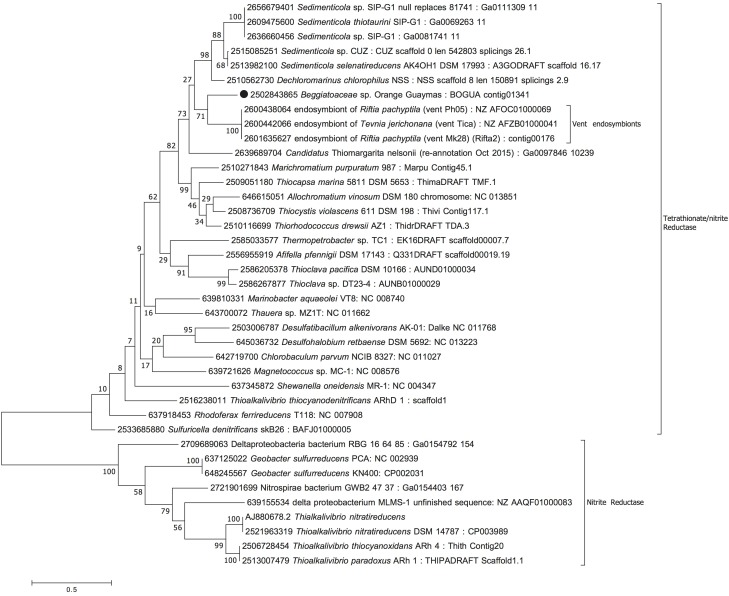
Maximum Likelihood phylogeny for candidate octaheme nitrite/tetrathionate reductase (BOGUAY_2386), based on aligned protein sequences obtained by translating the gene. Scale bar, bootstrap and taxon label annotation are the same as in Figure 3.

We inferred phylogenetic trees based on amino acid sequences of the multifunctional orange octaheme cytochrome in BOGUAY_0691 (Figure 3), the candidate nitrite reductase NirS in BOGUAY_2967 (Figure 4), the octaheme cytochrome *c* reductase, BOGUAY_2386 (Figure 5), and their homologs pulled from partial or complete genomes, in particular sulfur-oxidizing Gammaproteobacteria. The alignments were anchored in conserved CxxCH cytochrome binding sites and are included as Supplementary Figure 1 (BOGUAY_0691), 2 (BOGUAY_2967), and 3 (BOGUAY_2386).

The multifunctional orange octaheme cytochrome, consistently aligned around eight heme-binding sites (Supplementary Figure 1), formed a well-supported lineage (100% bootstrap support; Figure 3) with homologs from large, vacuolated, nitrate-accumulating and nitrate-reducing sulfur bacteria of the genus *Thiomargarita* (Flood et al., 2016; Winkel et al., 2016). A sister lineage of the multifunctional orange octaheme cytochrome was found in the bacterial SUP05 clade, recently cultured as *Candidatus* Thioglobus autotrophicus, a nitrate-respiring, nitrite-producing autotrophic sulfur oxidizer that thrives in marine oxygen minimum zones and stratified water columns (Anantharaman et al., 2013; Shah et al., 2017; Figure 3). Interestingly, *Candidatus* Thioglobus autotrophicus did not grow with nitrite as sole electron acceptor, suggesting that the octaheme cytochrome homolog of this bacterium does not function as a respiratory nitrite reductase. Further, *Candidatus* Thioglobus autotrophicus assimilated ammonium for growth but did not use it for anammox-like conproportionation with nitrite (Shah et al., 2017).

The NirS candidate protein (Figure 4) in the orange Guaymas filaments has homologs in a wide range of Gammaproteobacteria and other bacteria (Supplementary Figure 2); it forms an independently branching lineage parallel to the predicted NirS versions of vacuolated, nitrate-accumulating sulfur bacteria of the genus *Thiomargarita* (Flood et al., 2016; Winkel et al., 2016) and of the sulfur-oxidizing, nitrate-reducing filamentous bacterium *Thioploca ingrica* (Kojima et al., 2015). These homologs were not available in databases (JGI and GenBank) when this *nirS* candidate gene was originally described in the orange Guaymas *Beggiatoaceae*, with the consequence that its identification had remained tentative (MacGregor et al., 2013a; Figure 4). In *Thiomargarita* spp. and in *Thioploca ingrica*, all components of a complete denitrification pathway were found, together with the genes for dissimilatory (*Thiomargarita* spp.) and assimilatory (*Thioploca ingrica*) reduction to ammonium (Winkel et al., 2016). In contrast to *Thiomargarita* spp. and *Thioploca ingrica*, the genome of the orange Guaymas *Beggiatoaceae* indicates that the denitrification pathway is incomplete; it appears to lead to the formation of N_2_O but not dinitrogen (MacGregor et al., 2013a). Since the predicted orange Guaymas NirS does not form a well-supported clade with its counterparts in *Thiomargarita* and *Thioploca* (the relevant node has only 13% bootstrap support), and its gene neighborhood does not indicate any linkage to nitrate or nitrite respiration (Supplementary Table 3), it is likely that the orange Guaymas *Beggiatoaceae* have obtained this gene laterally, which is consistent with the absence of other *nir* genes in this genome (Schutte et al., 2018).

The candidate octaheme cytochrome c tetrathionate/nitrite reductase (BOGUAY_2386) was most closely related to homologs from sulfur-oxidizing bacterial endosymbionts of the chemosynthetic, sulfur-dependent vent tube worms *Riftia pachyptila* and *Tevnia jerichonana* (Gardebrecht et al., 2012), from the nitrate-reducing, sulfur-oxidizing vacuolated bacterium *Candidatus* Thiomargarita nelsonii (Winkel et al., 2016), and from the nitrite-, nitrate-, and selenite-reducing, aromatics-degrading bacterial genus *Sedimenticola* (Narasingarao and Häggblom, 2006; Figure 5). Within the trophosome, the symbiont host tissue in *Riftia pachyptila*, nitrite was reduced to ammonia whereas dinitrogen was not produced (Girguis et al., 2000). The nitrite-reducing, ammonia-producing function could be linked to the endobiont’s octaheme enzyme; the nitrite reduction product ammonia provides a symbiont-derived nitrogen source – particularly relevant in nitrogen-limited vent habitats – that can be assimilated by *Riftia* (Robidart et al., 2011). BOGUAY_2386 was also homologous to the structurally well-studied octaheme cytochrome tetrathionate reductase in *Shewanella oneidensis* (Mowat et al., 2004), which occupied a basal position in this phylogenetic branch of octaheme cytochrome *c* reductases (Figure 5). Finally, the nitrite reductase in *Thioalkalivibrio nitratireducens* (Tikhonova et al., 2006) and its relatives formed a phylogenetic sister lineage to the *Shewanella* branch (Figure 5). The protein alignment demonstrates the evolutionary divergence between the *Shewanella* tetrathionate reductase and the *Thioalkalivibrio* nitrite reductase lineages; yet they share the eight heme-binding sites that are characteristic for this enzyme type (Supplementary Figure 3; Tikhonova et al., 2012). As a caveat, substrate preference (nitrite vs. tetrathionate) and enzyme function should not be predicted based on phylogenetic position alone; other factors, such as genomic and physiological context, and changing environmental selection factors during the evolutionary trajectory of this enzyme family, are also likely to influence the final substrate preference and activity.

### Activities of Expressed Enzymes

**FIGURE 6:**
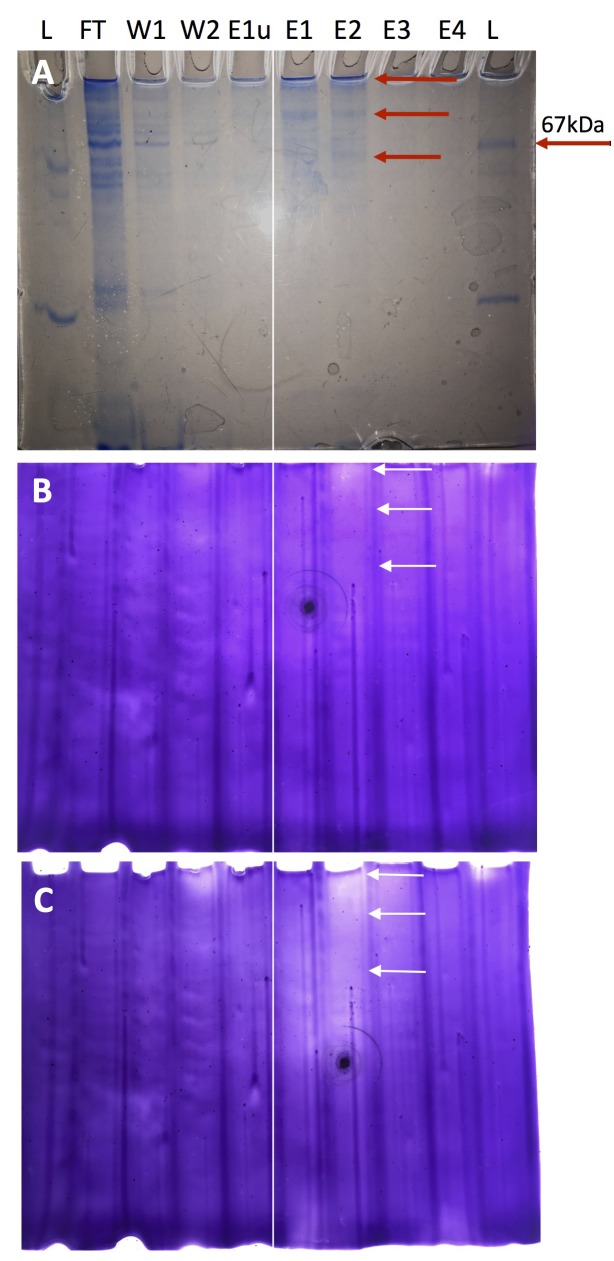
In-gel nitrite reduction assay for expressed protein of BOGUAY_2386. **(A)** Coomassie total protein stain; the arrow identifies the 67 kda marker protein. **(B)** Nitrite reduction gel after 2 h development of the Methyl Viologen (MV) destaining reaction in anaerobe chamber followed by 1 min exposure to atmospheric oxygen. **(C)** Nitrite reduction gel after 2 h development in anaerobe chamber and 30 min exposure to atmospheric oxygen. The expected molecular weight for the monomer is 65.1 kba, which matches the position of the lowermost band close to the 67 kba ladder protein **(A)**. Additional bands of higher molecular weight that show nitrite-reducing enzymatic activity are presumably dimers and – near the top of the lane – non-migrating multimers that may reflect partial hydrophobicity of the protein. The monomer and the multimers are marked with arrows in all three panels. L, Ladder; FT, Flow through of cell lysate; W1, Wash 1; W2, Wash 2; E1u, Elution 1 of uninduced protein purification; E1, first elution of induced protein purification; E2, second elution of induced protein purification; E3, third elution 3 of induced protein purification; E4, Fourth elution 4 of induced protein purification. For orientation, a thin white line is placed between the E1u and E1 lane in all gels.

When the cell extracts and purified proteins were tested *in vitro*, the strongest *in vitro* nitrite reductase activity for a purified enzyme was found for the BOGUAY_2386 enzyme (Table 3). In-gel tests with nitrite as electron acceptor and sodium dithionite as electron donor in polyacrylamide gels demonstrated that this candidate gene retained its activity (Figure 6). The inferred amino acid sequence is predicted by SignalP to have N- and C-terminal transmembrane helices, but is otherwise predicted to be soluble. This would be consistent with preserved *in vitro* and in-gel nitrite-reducing activity of the partially purified protein, outside of the membrane context (Table 3), and is also consistent with the three-dimensional protein structure inferred by protein threading (Supplementary Figure 4A).

**Table 3:** Spectrophotometric nitrite reductase enzymatic assays of cell lysates and partially purified proteins.

ORF	Gene expression sample	Activity
BOGUAY_2386	ONR E2-6-14-2016	1.287 ± 0.0016
	Purified protein, second elution	
BOGUAY_2967	nirS cell lysate + IPTG	2.624 ± 0.64
BOGUAY_2967	nirS cell lysate – IPTG	0.0009 ± 0.0008
BOGUAY_0691	Orange Protein E1	0.0144 ± 0.0025
	Purified protein	
BOGUAY_0691	Orange Protein E2	0.0028 ± 0.0014
	Purified protein	
BOGUAY_0691	Original orange protein sample (MacGregor et al., 2013b)	0.052 ± 0.025
BOGUAY_0691	Partially purified sample (MacGregor et al., 2013b)	0.31 ± 0.1


*Activity is given in μmol min^-*1*^ mg protein^-*1*^. The negative controls for BSA and the cell lysate of the expression strain pET22b showed activities of 0.0005 and 0.0003 μmol min^-*1*^ mg protein^-*1*^, respectively. For all assays, activities are shown as units ± standard deviation (*n* ≥ 3).*

Since the phylogenetic affiliation of BOGUAY_2386 to the *Shewanella* octaheme cytochrome tetrathionate reductase (Mowat et al., 2004) suggested a shared function, we tested tetrathionate reductase activity *in vitro*, and found an activity up to 93 μmoles tetrathionate reduced per minute per mg of purified recombinant BOGUAY_2386 protein (Table 4), significantly higher than the observed nitrite reductase activity of the purified recombinant protein (Table 3). The purified recombinant protein showed higher activities than the corresponding cell lysates of the BOGUAY_2386 expressing *E. coli* (Table 4). In this reaction, two electrons reduce one molecule tetrathionate (S_4_O_6_^2-^) to two molecules of thiosulfate (S_2_O_3_^2^). Based on the well-studied tetrathionate reduction pathway in the bacterium *Salmonella enterica* serovar *typhimurium*, this reaction could have its physiological context and rationale in the enzymatic reduction of partially oxidized sulfur intermediates to hydrogen sulfide; the tetrathionate reduction product thiosulfate is reduced with a two-electron transfer step to sulfite and sulfide, and sulfite is reduced in a six-electron transfer step to sulfide (Barrett and Clark, 1987; Price-Carter et al., 2001). The genomic context of putative thiosulfate and tetrathionate reductase gene subunits just downstream of BOGUAY_2386 (Supplementary Table 3) suggests the possibility that the orange *Beggiatoaceae* can reduce incompletely oxidized sulfur compounds such as thiosulfate, tetrathionate and sulfite, analogous to sulfur-reducing capacity shown previously for heterotrophic freshwater *Beggiatoa* spp. (Nelson and Castenholz, 1981; Schmidt et al., 1987) as well as hydrogenotrophic marine *Beggiatoa* spp. (Schwedt et al., 2011; Kreutzmann and Schulz-Vogt, 2016). Putative genes coding for the key enzyme of sulfite reduction, dissimilatory sulfite reductase, have previously been found on BOGUAY contigs; dsrAB was identified on contigs BOGUAY_01191_1510 and 1511. Potential genes for other dsr subunits, except DsrT, were found on contigs BOGUAY_01191_1500 to 1508 (Supplementary Table 1 in MacGregor et al., 2013a). As a caveat, these Dsr subunits were annotated as the reverse, oxidative version of dissimilatory sulfite reductase (rDSR) by MacGregor et al. (2013a); ultimately the reductive vs. oxidative directionality of this enzyme in the orange *Beggiatoaceae* remains to be determined experimentally. Overall, the ability to reduce sulfur compounds would enable the Guaymas *Beggiatoaceae* to adapt their energy metabolism to reducing conditions when neither oxygen nor nitrate are sufficiently available; such conditions may occur when pulses of hydrothermal fluids rich in sulfur compounds are flushing surficial Guaymas Basin sediments (McKay et al., 2016; Teske et al., 2016).

**Table 4:** Spectrophotometric tetrathionate reductase enzymatic assays of cell lysates and partially purified proteins.

ORF	Gene expression sample	Activity
BOGUAY_2386	ONR cell lysate + IPTG	0.702 ± 0.346
BOGUAY_2386	2386 E1-7-15-17, purified protein first elution	93.391 ± 37.739
BOGUAY_2386	ONR E3-9-28-2017	21.939 ± 9.819
	Purified protein, third elution	


*Activity is given in μmol min^-*1*^ mg protein^-*1*^. The negative controls for BSA showed activities of 0.195 μmol min^-*1*^ mg protein^-*1*^. For all assays, activities are shown as units ± standard deviation (*n* ≥ 3).*

The *nirS* candidate gene (BOGUAY_2967) product showed *in vitro* nitrite reductase activity only in the cell lysate of transformed cells (Table 3), whereas measurements for purified proteins showed no activity (results not shown). During in-gel assays, the candidate NirS protein denatured completely when entering the gel, and showed no activity. This striking contrast to high *in vitro* activity of the NirS cell lysate suggests that the protein may be membrane-associated or membrane-embedded. However, the NirS candidate gene does not encode any transmembrane helices (Supplementary Figure 4B); therefore, a more likely interpretation is that the host cell lysate provides unidentified cofactors or other essential subunits of the holoenzyme that would be lost from purified proteins.

The partially purified orange octaheme cytochrome (BOGUAY_0691) protein showed considerably reduced activity, by ca. two orders of magnitude (Table 3), compared to the original microbial mat sample (frozen at –80°C during transport to the home lab) and the partially purified orange protein after ammonium sulfate precipitation from solution (MacGregor et al., 2013b). The orange protein did not show any in-gel activity for nitrite reduction (results not shown). Membrane association does not explain these results, since the orange protein does not contain any transmembrane anchor motifs (Supplementary Figure 4C) and is predicted to be soluble, as found during its original isolation (MacGregor et al., 2013b). It appears likely that reducing conditions are essential for retaining activity, as previous tests were performed in the presence of beta-mercaptoethanol (MacGregor et al., 2013a). Although the need for an unknown cofactor cannot be ruled out entirely, based on shipboard observations we infer that this protein is particularly sensitive to oxidative damage. Freshly collected individual orange *Beggiatoaceae* filaments from Guaymas Basin were examined in the shipboard lab submerged in glass petri dishes with fully oxic seawater under a dissection scope; these specimens repeatedly lost their orange color after ca. 30 to 60 min of exposure, whereas aggregated filament clusters did not fade (A. Teske, unpublished shipboard observations).

## Discussion

### Physiological Roles of Candidate Genes

The finding that all three gene products reduce nitrite *in vitro* leads to the question of their specific physiological roles, since it is unlikely that these enzymes function redundantly in the same manner within the same organism. We propose the following differentiation.

The candidate *nirS* gene product is likely to function as a nitrite reductase that receives its substrate from nitrate reduction; the most likely end product of nitrate/nitrite reduction would be nitrous oxide, since no nitrous oxide reductase could be found in the orange *Beggiatoaceae* genome (MacGregor et al., 2013a,b). Since the orange *Beggiatoaceae* possessed *nirS* and *nirE* but lacked other identifiable *nir* genes (Schutte et al., 2018), we add the caveat that this *nirS* candidate gene product may require unidentified cofactors to be functional, as suggested by the observation that enzymatic activity was found in whole extracts of transformed *E. coli* cells but never after purification (Table 2).

The tetrathionate and nitrite-reducing octaheme cytochrome c reductase is the only protein that showed robust nitrite-reducing activity *in vitro* after protein purification and in polyacrylamide gels. Based on homology to the ammonia-producing *Shewanella oneidensis* version of this enzyme (Mowat et al., 2004; Mowat and Chapman, 2005; Atkinson et al., 2007; Einsle, 2011) it is a candidate for catalyzing dissimilatory nitrite reduction to ammonia, the *in vivo* product of nitrate reduction in orange Guaymas *Beggiatoaceae* (Schutte et al., 2018). The phylogenetic similarity of the BOGUAY_2386 gene product to the *Shewanella* version is also reflected in the shared tetrathionate reductase activity of the purified proteins. Comparison with the dihaem cytochrome *c* tetrathionate reductase, TsdA, from the microaerophilic gut bacterium *Campylobacter jejuni*, shows that the BOGUAY_2386 enzyme has a higher rate of tetrathionate reductase activity than cells of whole *C. jejuni* cell suspensions that are *tsdA*+ (0.08 μmol min^-1^ mg cell protein^-1^; Liu et al., 2013), but less than purified recombinant TsdA from *C. jejuni* (1316 μmol min^-1^ mg cell protein^-1^ for the wild type; Kurth et al., 2016). In *C. jejuni*, tetrathionate is considered an alternate electron acceptor to be used under anoxic conditions (Liu et al., 2013). Similarly, the BOGUAY_2386 enzyme confers the ability to use alternate electron acceptors in addition to nitrate and nitrite, including partially oxidized sulfur species such as tetrathionate and thiosulfate. Such electron acceptors could be generated by incomplete oxidation of hydrothermal sulfide under microoxic conditions at the sediment surface, the same process that leads to sulfur and polysulfide enrichment at the sediment surface (Teske et al., 2016). Given the preference of the orange *Beggiatoaceae* for the central areas of hydrothermal hot spots where the strongest upflow of sulfur-rich fluids coexists with surficial seawater inmixing, the BOGUAY_2386 enzyme could play a significant role in the reduction of partially oxidized sulfur compounds in its natural habitat.

The multifunctional orange octaheme cytochrome remains intriguing. This enzyme could be interpreted as an alternate nitrite reductase that complements the activity of the *nirS* gene product or the nitrite/tetrathionate reductase, but it could play an additional or alternate role in detoxification of excess nitrite and other nitrogenous compounds (MacGregor et al., 2013b). Since it is sensitive to oxidizing conditions, and requires beta-mercaptoethanol to test positively during in-gel activity assays (MacGregor et al., 2013b), it is possible that this enzyme is specifically adapted to reducing conditions generated by pulses of hydrothermal flow in hydrothermal sediments (McKay et al., 2016).

### Ecological Rationale for Different Nitrite and Nitrate Reduction Pathways

Alternate genes and pathways of nitrite reduction and tetrathionate reduction could represent a response to high sulfide concentrations and reflect the need to use potentially limiting electron acceptors most efficiently. Increased hydrothermal flow and higher sulfide concentrations in the surficial sediments characterize the habitat preference of the orange *Beggiatoaceae* in the center of hydrothermal hot spots and distinguish it from the non-pigmented *Beggiatoaceae* mats on the periphery of hydrothermal sediments (McKay et al., 2012). Under strongly reducing conditions when sulfide is abundant, but nitrite and nitrate are limiting, reduction to ammonia consumes more electrons than reduction to N_2_ or nitrous oxide. Indeed, comparative nitrate reduction experiments have shown that the orange Guaymas *Beggiatoaceae* produced ammonia but not N_2_, whereas the unpigmented Guaymas *Beggiatoaceae* generated primarily N_2_ and produced ammonia only under conditions of oxidant limitation (Schutte et al., 2018).

Interestingly, diverse sulfur-oxidizing nitrate-reducing bacteria show habitat-dependent, ecologically fine-tuned stoichiometries of N-S redox reactions. The marine benthic sulfur oxidizing bacterium *Thioploca* reduces nitrate to ammonium, an eight-electron transfer reaction, in order to maximize sulfide removal and to maintain low, non-toxic sulfide concentrations in organic-rich sediments with very high sulfate reduction rates (Hüttel et al., 1996; Otte et al., 1999). In contrast, nitrate or nitrite reduction that terminates at N_2_O accepts only four electrons per nitrate; such a mechanism would be possible when the electron donor sulfide is less abundant and does not have to be consumed with maximum efficiency. Once sulfide becomes limiting and nitrate is consistently abundant, the electron acceptor can be used for single-step reduction to nitrite. For example, field observations in oxygen minimum zones offshore Chile have linked sulfide oxidation to the reduction of nitrate to nitrite (Canfield et al., 2010); the dominant pelagic sulfur oxidizer identified in this study, SUP05, was later cultivated as *Candidatus* Thioglobus autotrophica, and shown to reduce nitrate to nitrite (Shah et al., 2017). In this view of sulfide-dependent nitrate reduction, the stoichiometry and preferred pathway of nitrate reduction adapts to the availability and abundance of sulfide (Teske, 2010).

## Conclusion

Sulfide toxicity is an important factor that may have selected against classical denitrification and instead for alternate nitrite reduction pathways in orange Guaymas *Beggiatoaceae*. High sulfide concentrations impact denitrification rates in Guaymas Basin hydrothermal sediments (Bowles et al., 2012). Sulfide additions decreased the relative proportion of denitrification to N_2_ compared to overall nitrate consumption, and apparently shifted nitrate reduction from denitrification to other dissimilatory pathways, such as dissimilatory reduction to ammonia (Bowles et al., 2012). Responding to sulfide stress by dissimilatory reduction of nitrate and nitrite to ammonia, by shutting down (or not even possessing) a classical sulfide-sensitive denitrification pathway, or by expressing tetrathionate/thiosulfate-reducing enzymes that allow the reduction of these partially oxidized sulfur species (with low-molecular-weight organic substrates or hydrogen as electron donors), would provide a selective advantage for the orange *Beggiatoaceae*, and explain their consistent enrichment in the center of mat-covered hydrothermal hot spots where the upward flow of sulfidic fluids toward the sediment surface is most intense (McKay et al., 2012). In contrast, the large unpigmented *Beggiatoaceae* that dominate the margins of hydrothermal hot spots in Guaymas Basin retain a classical denitrification pathway that reflects conditions of lower hydrothermal flow, lower sulfide supply and gradually increasing oxidizing conditions (Schutte et al., 2018). To conclude, different nitrite-reducing enzymes with multiple functionalities reveal subtle environmental adaptations of *Beggiatoaceae* to their hydrothermal mat habitat that provide rewarding targets for further investigation.

## Data Availability

Publicly available datasets were analyzed in this study. This data can be found at http://www.jgi.doe.gov.

## Author Contributions

AB developed the experimental plan, performed the experiments, constructed the protein alignments, and inferred the phylogenetic trees. BM provided the *Beggiatoaceae* genome contigs and expertise on *Beggiatoaceae* genomics. AT initiated the project and wrote the manuscript, with input from all authors.

## Conflict of Interest Statement

The authors declare that the research was conducted in the absence of any commercial or financial relationships that could be construed as a potential conflict of interest.

## Footnotes

1 http://nc2.neb.com/NEBcutter2/

2 http://www.cbs.dtu.dk/services/SignalP/

3 http://www.pirx.com/iMol/index.shtml

4 http://www.jgi.doe.gov

5 http://www.jgi.doe.gov
