# Supplementary material for: Identification, Expression and Activity of Candidate Nitrite Reductases From Orange Beggiatoaceae, Guaymas Basin
Source: Front Microbiol. 2019 Mar 29;10:644. doi: 10.3389/fmicb.2019.00644 (PMC6449678; doi:10.3389/fmicb.2019.00644)
Supplement: Supplementary file 1 [file Data_Sheet_1.PDF]

Supplementary Material

to

Identification, Expression and Activity of Candidate Nitrite Reductases from orange *Beggiatoaceae*, Guaymas Basin

by Andrew Buckley, Barbara MacGregor, Andreas Teske

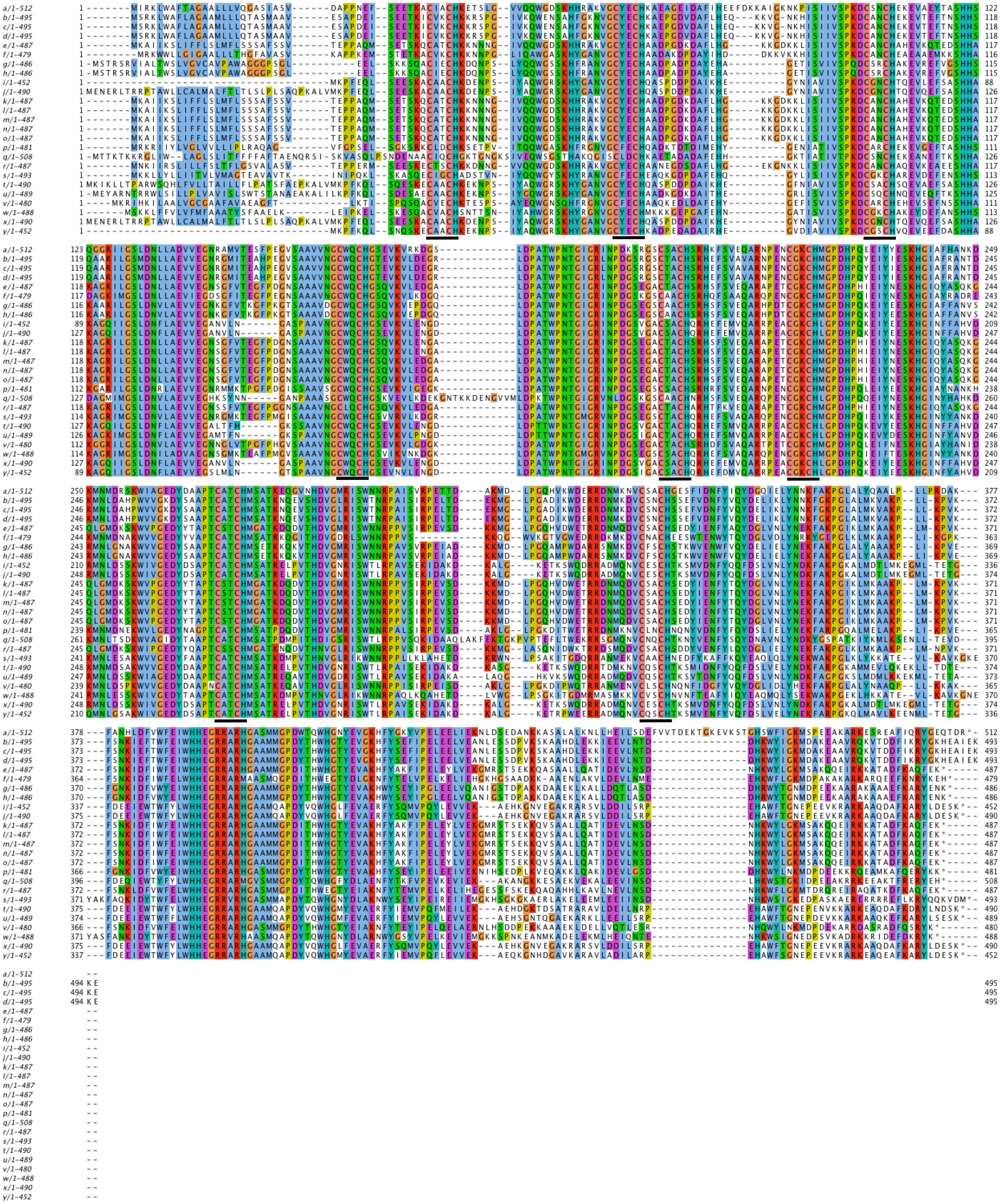

Supplementary Figure 1. Multiple sequence amino acid alignment of orange octaheme cytochrome (BOGUAY_0691) and similar translated nucleotide sequences. Eight

potential heme-binding sites (CxxCH/K) were found, and are underlined. Homologs of the BOGUAY sequences (*) were identified by BLASTP searches of the IMG/ER and NCBI databases. Gene numbers for genome-derived sequences can be found in IMG/ER (http://www.jgi.doe.gov); Genbank Accession numbers may be alternatively used. Gene annotation legend: a*, 2502838749 *Beggiatoa* sp. Orange Guaymas; b, 2639695019 Candidatus Thiomargarita nelsonii Ga0097846 11237; c, 2639690618 *Candidatus* Thiomargarita nelsonii Ga0097846 10423; d, 2601778522 Thiomargarita nelsonii bud S10 Ga0063879 1005; e, 2647705483 *Candidatus* Thioglobus sp. EF1 Ga0100952 11; f, 2601634560 endosymbiont of *Riftia pachyptila* (vent Mk28) (Rifta2); g, 2716626204 *Lentisphaerae* bacterium RIFOXYB12 65 16 Ga0156531 1059; h, 2713279123 *Lentisphaerae* bacterium RIFOXYA12 64 32 Ga0156402 1279; i, 2546700336 *Vibrio fluvialis* I21563 ASXT01000041; j, 2525524676 *Vibrio sp. ZOR0035* Vib35DRAFT1 NODE 13 len 60483 cov 56 502224.12; k, 2528257581 SUP05 cluster bacterium AB-750I15AB-904; l, 2528261999 SUP05 cluster bacterium AB-750K23AB-904; m, 2527562523 SUP05 cluster bacterium AB-755 A05D07; n, 2528248240 SUP05 cluster bacterium AB-750C14AB-904; o, 2528265835 SUP05 cluster bacterium AB-750M18AB-904; p, 2721786119 *Omnitrophica WOR 2* bacterium GWA2 45 18 Ga0154332 122; q, 2721822901 *Bacteroidetes* bacterium GWA2 31 9: Ga0154347 1241; r, 2701081706 *Oceanospirillales* bacterium SCGC AD-311-B11v3 Ga0138937 105; s, 2730372430 *Nitrospirae* bacterium JdFR-81 Ga0180893 134; t, 2714139077 *Photobacterium ganghwense* DSM22954 Ga0124608 1005; u, 640935336 *Shewanella sediminis* HAW-EB3 NC 009831; v, 2617265124 *D.pulchra* bleached metagenome bin377 Ga0073125 1093; w, 2728920477 *Nitrospirae* bacterium HCH-1; x, 2721272550 *Vibrio fluvialis* ATCC33809 Ga0174860 12; y, 647235545 *Grimontia hollisae* CIP101886 NZ ADAQ01000010.

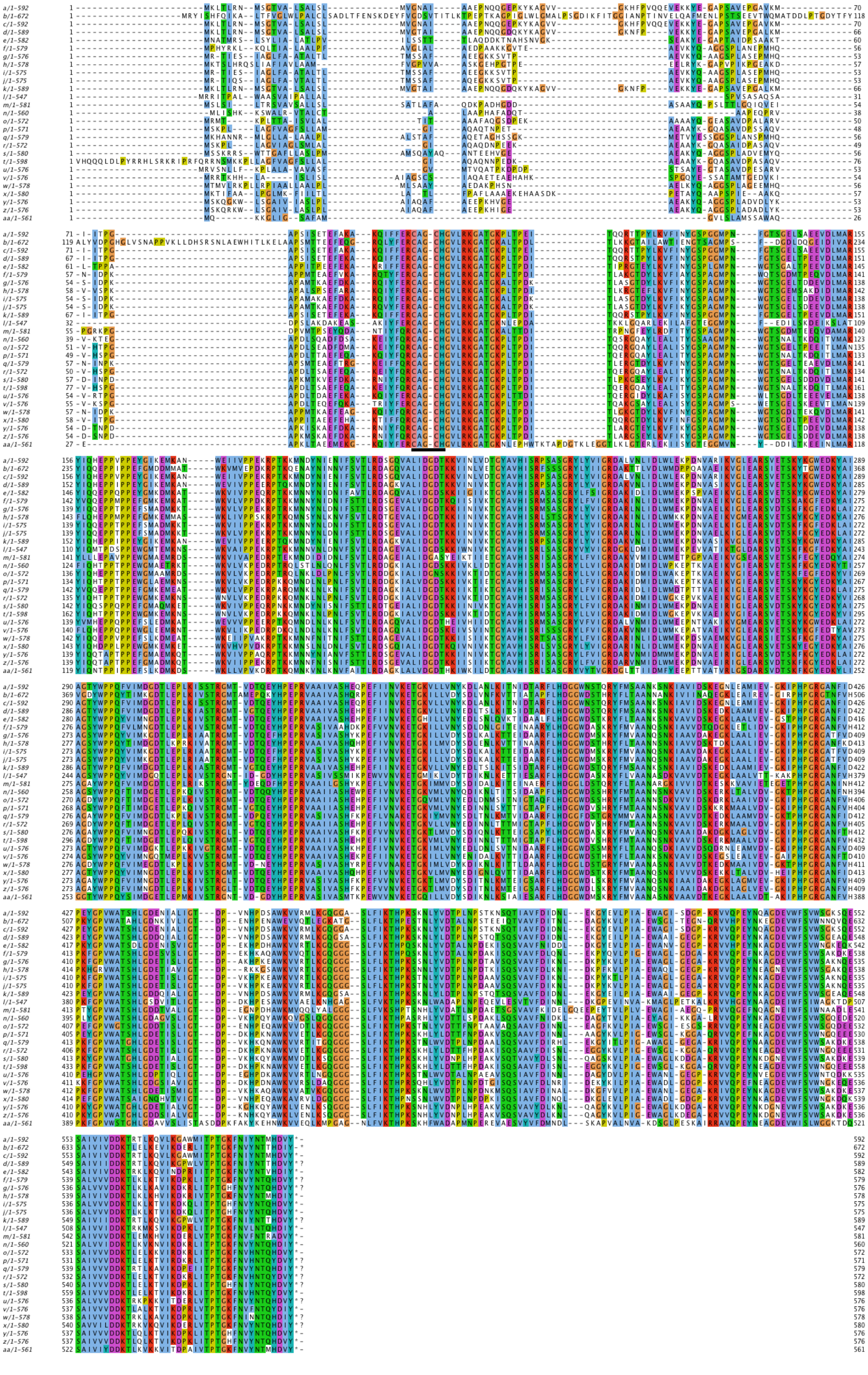

Supplementary Figure 2. Multiple sequence amino acid alignment of candidate NirS protein (BOGUAY_2967) and similar translated nucleotide sequences. Potential heme-binding sites (CxxCH/K) are underlined. Homologs of the BOGUAY sequences (*) were identified by BLASTP searches of the IMG/ER and NCBI databases. Gene numbers for genome-derived sequences can be found in IMG/ER (http://www.jgi.doe.gov); Genbank Accession numbers may be alternatively used when available. Gene annotation legend: a, 2236869020 *Thiomargarita sp. Thio36*: Thi036DRAFT; b*, 2502841072 *Beggiatoa* sp. Orange Guaymas: BOGUA contig00500; c, 2639701232 *Candidatus* Thiomargarita nelsonii (re-annotation Oct 2015); d, 2639695833 *Candidatus* Thiomargarita nelsonii (re-annotation Oct 2015): Ga0097846 11335; e, 2621211924 *Thioploca ingrica*: Ga0060138 11; f, 2623356007 Gammaproteobacterium bin V90401B4 Ga0074589: Ga0074589 1003; g, 2557376323 *Candidatus* Accumulibacter sp. BA-91; h, 2656887058 *Acidithiobacillales* bacterium SG8 45: Ga0111141 118; i, 2557361812 *Candidatus* Accumulibacter sp. SK-02; j, 2557354053 *Candidatus* Accumulibacter sp. SK-01; k, 2601782927 *Thiomargarita nelsonii* bud S10 Ga0063879: Ga0063879 1104; l, AJ224912.1 *Azospirillum brasilense* Sp7 DSM 1690; m, AJ224911.1 *Roseobacter denitrificans* ATCC 33942T; n, AF114792.1 *Pseudomonas fluorescens;* o, 2619824534 *Pseudomonas bauzanensis*: Ga0066784 101; p, 2632721459 *Pseudomonas stutzeri* YC-YH1: Ga0080851 106; q, 646688790 *Sideroxydans lithotrophicus* ES-1; r, 2518856877 *Pseudomonas stutzeri* Ursing AN10: CP003677; s, 637607693 *Azoarcus sp. EbN*: NC 006513; t, 651021683 *Pseudomonas stutzeri* ATCC 17588; u, 2516837226 *Marinobacter salsuginis* SD-14B : Msal Contig_88; v, 2520613139 *Hahella ganghwensis* DSM 17046; w, 2586350263 *Herminiimonas sp. CN;* x, 2711457500 Gammaproteobacteria bacterium: Ga0156034 1087; y, 2681855052 *Azoarcus tolulyticus;* z, 2518919693 *Azoarcus toluclasticus;* aa, 643699541 *Thauera aminoaromatica.*

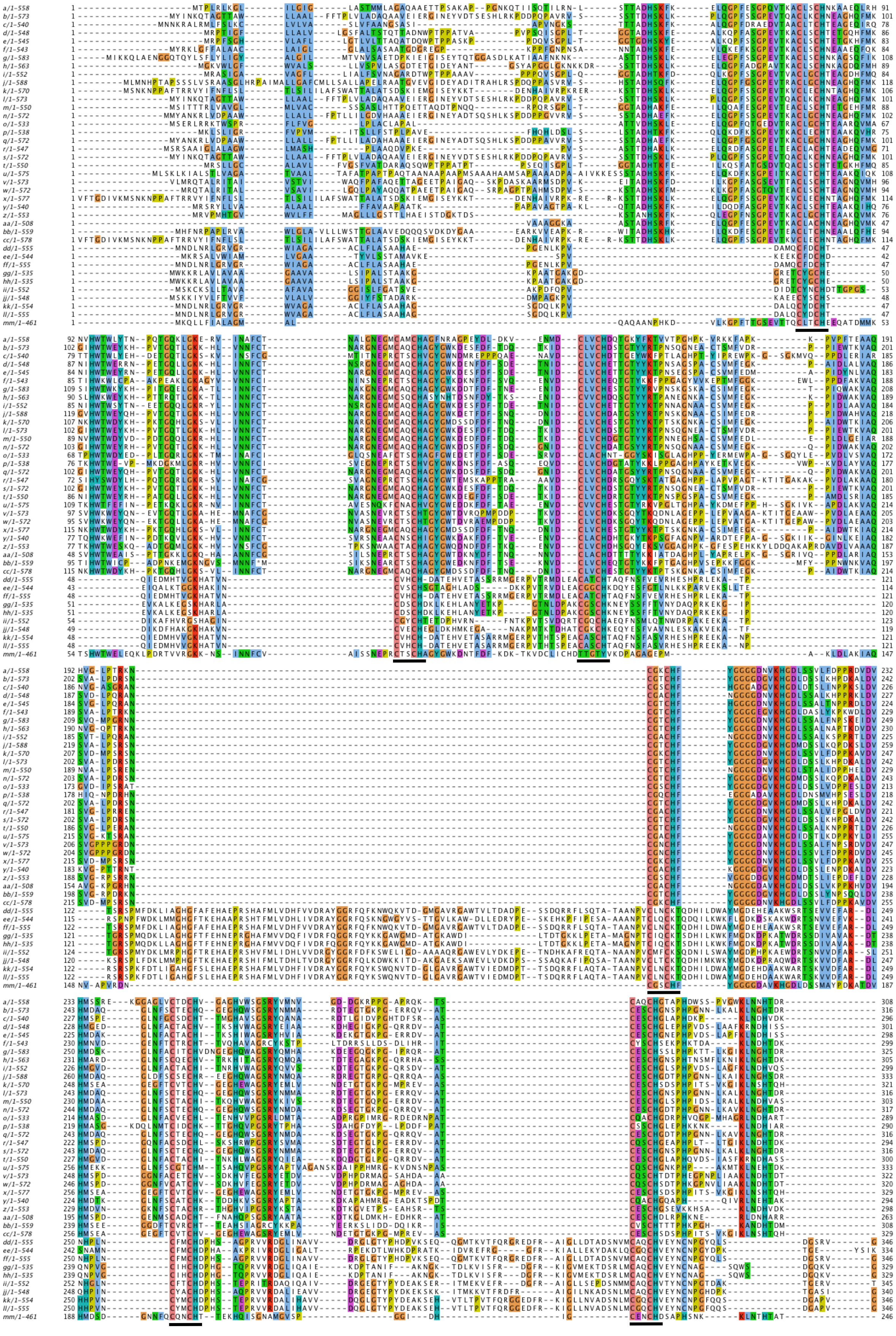

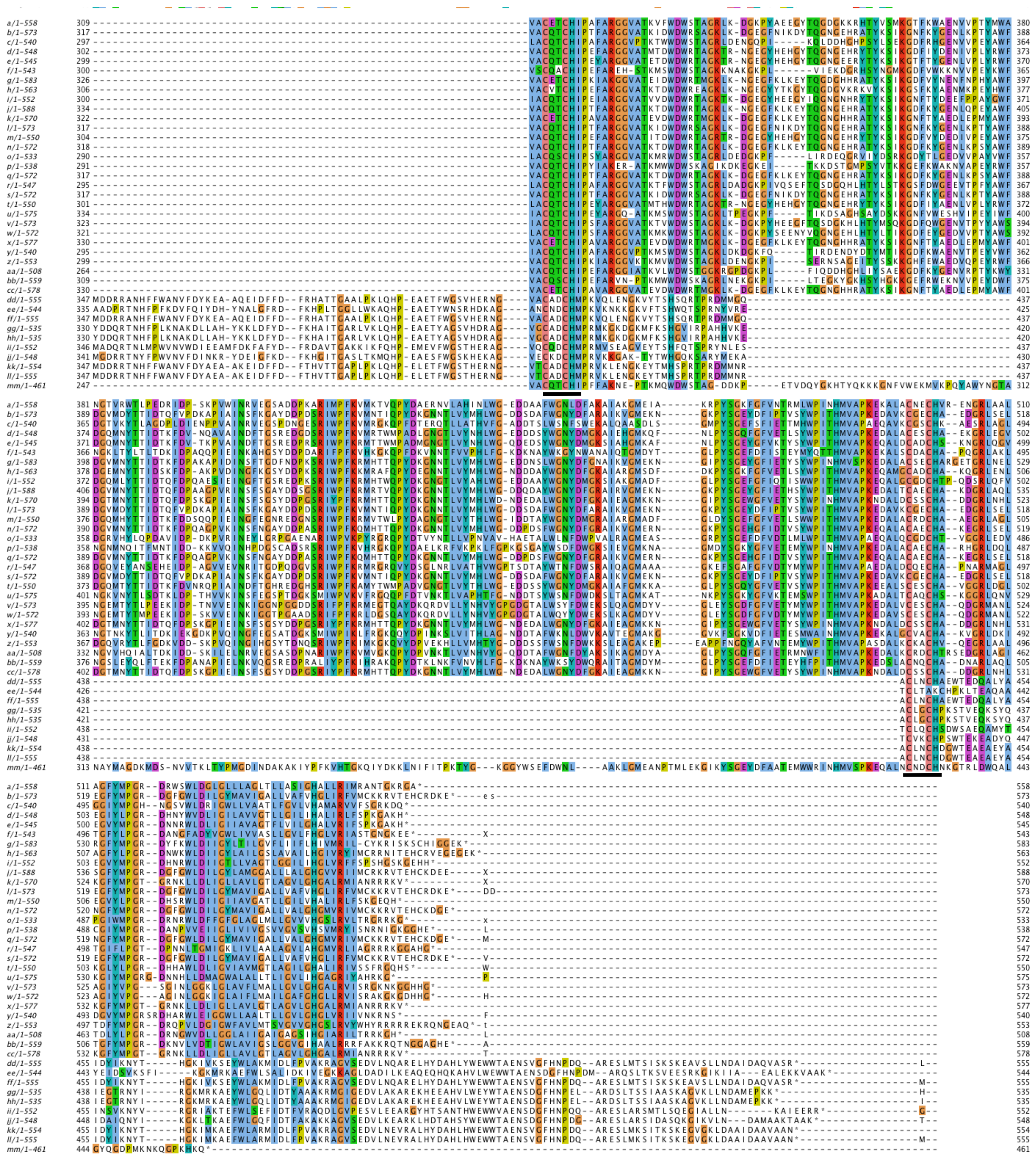

Supplementary Figure 3. Multiple sequence amino acid alignment of candidate ammonia-producing octaheme cytochrome c reductase (BOGUAY_2386) and similar translated nucleotide sequences. Potential heme-binding sites (CxxCH/K) are underlined. Homologs of the BOGUAY sequences (*) were identified by BLASTP searches of the IMG/ER and NCBI databases. Gene numbers for genome-derived sequences can be found in IMG/ER (http://www.jgi.doe.gov); Genbank Accession numbers may be alternatively used when available. Gene annotation legend: a, 2585033577 *Thermopetrobacter sp. TC1;* b, 2636660456 *Sedimenticola sp SIP-G1*

Ga0081741 11; c, 639810331 *Marinobacter aquaeolei* VT8 NC008740; d, 2508736709 *Thiocystis violascens* 611 DSM198; e, 2510116699 *Thiorhodococcus drewsii* AZ1; f, 2503006787 *Desulfatibacillum alkenivorans* AK-01 NC011768; g*, 2502843865 *Beggiatoa* sp. Orange Guaymas; h, 2639689704 *Candidatus* Thiomargarita nelsonii Ga009784610239; i, 2509051180 *Thiocapsa marina* 5811 DSM5653; j, 2510562730 *Dechloromarinus chlorophilus* NSS; k, 2600442066 endosymbiont of *Tevnia jerichonana* (vent Tica) NZAFZB01000041; l, 2656679401 *Sedimenticola sp. SIP-G1* Ga011130911; m, 2510271843 *Marichromatium purpuratum* 987; n, 2515085251 *Sedimenticola sp. CUZ;* o, 2516238011 *Thioalkalivibrio thiocyanodenitrificans* ARhD1; p, 642719700 *Chlorobaculum parvum* NCIB 8327 NC011027; q, 2513982100 *Sedimenticola selenatireducens* AK4OH1 DSM 17993; r, 2556955919 *Afifella pfennigii* DSM17143; s, 2609475600 *Sedimenticola thiotaurini* SIP-G1 Ga006926311; t, 646615051 *Allochromatium vinosum* DSM180 NC013851; u, 2533685880 *Sulfuricella denitrificans* skB26 BAFJ01000005; v, 2586205378 *Thioclava pacifica* DSM10166 AUND01000034; w, 2586267877 *Thioclava sp. DT23* 4 AUNB01000029; x, 2600438064 endosymbiont of *Riftia pachyptila* (vent Ph05) NZAFOC01000069; y, 637918453 *Rhodoferax ferrireducens* T118 NC007908; z, 639721626 *Magnetococcus sp. MC-1* NC008576; aa, 643700072 *Thauera sp. MZ1T* NC011662; bb, 645036732 *Desulfohalobium retbaense* DSM5692 NC013223; cc, 2601635627 endosymbiont of *Riftia pachyptila* (vent Mk28) (Rifta2); dd, AJ880678.2 *Thialkalivibrio nitratireducens;* ee, 2709689063 Deltaproteobacteria bacterium RBG166485 Ga0154792154; ff, 2521963319 *Thioalkalivibrio nitratireducens* DSM14787 CP003989; gg, 637125022 *Geobacter sulfurreducens* PCA NC002939; hh, 648245567 *Geobacter sulfurreducens* KN400 CP002031; ii, 639155534 delta proteobacterium MLMS 1 NZAAQF01000083; jj, 2721901699 *Nitrospirae* bacterium GWB24737 Ga0154403167; kk, 2506728454 *Thioalkalivibrio thiocyanoxidans ARh4;* ll, 2513007479 *Thioalkalivibrio paradoxus ARh1;* mm, 637345872 *Shewanella oneidensis MR-1* NC004347.

Supplementary Figure 4. Three-dimensional protein-threading models for candidate nitrite reductases**. A**) top left, BOGUAY_2386 protein threaded model generated by MUSTER with the highest z-score prediction of 14.586. **B**) bottom left, BOGUAY_2967 protein threaded model generated by MUSTER with the highest z-score prediction of 21.683. **C**) top right, BOGUAY_0691 protein threaded model generated by MUSTER with the highest z-score prediction of 9.735. Models were visualized with iMol software. All predicted protein structures are colored based on protein sequence position from N-terminal (blue) to C-terminal (red).

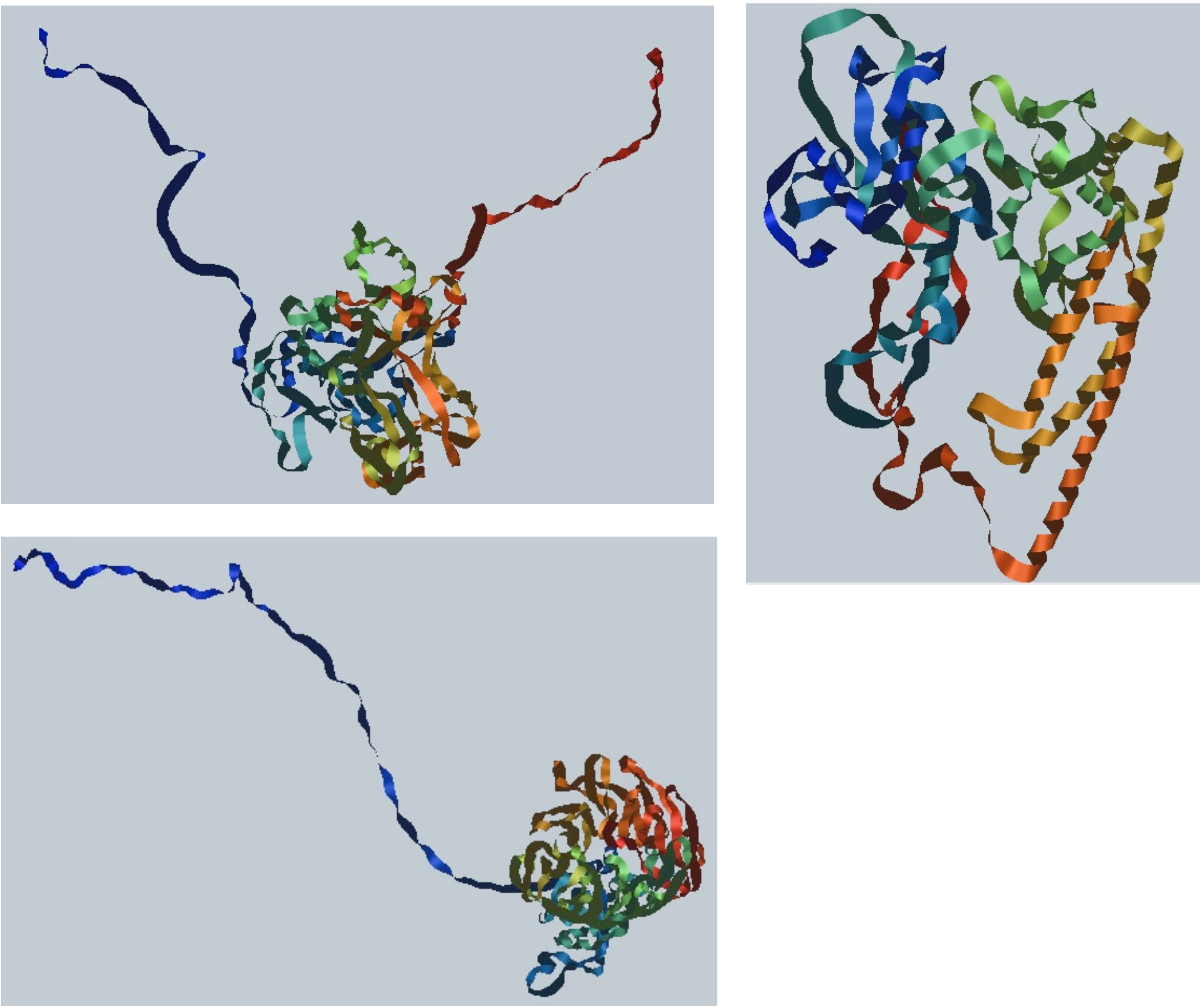

Supplementary Table 1. Plasmids used in this study.

Plasmid	Description
pCR-XL TOPO	Cloning vector. Kan^R (Thermo-Fisher, Waltham, MA)
pCR-2.1	Cloning vector. Kan^R (Thermo-Fisher, Waltham, MA)
pET22b	Expression vector. Amp^R (Gift from Quivey Lab, Univ. of Rochester)
pCRXL-2967ext	pCR-XL-TOPO with BOGUAY_2967 with flanking regions
pCRXL-2386ext	pCR-XL-TOPO with BOGUAY_2386 with flanking regions
pCR2.1-0651ext	pCR-2.1 with BOGUAY_0691 with flanking regions
pCR2.1-2967FR	pCR-2.1 with BOGUAY_2967
pCR2.1-2386FR	pCR-2.1 with BOGUAY_2386
pCR2.1-0691FR	pCR-2.1 with BOGUAY_0691
pCR2.1-2967FRRE	pCR-2.1 with BOGUAY_2967 with restriction sites
pCR2.1-2386FRRE	pCR-2.1 with BOGUAY_2386 with restriction sites
pCR2.1-0691FRRE	pCR-2.1 with BOGUAY_0691 with restriction sites
pET22b	Protein Expression vector (EMD Millipore, Billerica, MA). Amp^R.
pET22b-2967FRRE	pET22b with the inducible BOGUAY_2967 gene product
pET22b-2386FRRE	pET22b with the inducible BOGUAY_2386 gene product
pET22b-0691FRRE	pET22b with the inducible BOGUAY_0691 gene product

Supplementary Table 2. *E. coli* strains used in this study.

Strain Name	Strain Description
Oneshot® Top10 *E. coli*	Strain for transformation and propagation of PCR-cloning products. (Thermo Fisher, Waltham, MA)
BL21 ∂DE3	E. coli strain utilizes T7 promoter system for recombinant protein expression, carries lambda DE3 lysogen (Gift from Quivey Lab, Uni. of Rochester).
BL21 ∂DE3+ pET22B	BL21 ∂DE3 strain with the pET22b vector.
BL21 ∂DE3+ pET22B2967OGB	BL21 ∂DE3 strain with the pET22b vector expressing BOGUAY_2967 when induced with IPTG.
BL21 ∂DE3+ pET22B2386OGB	BL21 ∂DE3 strain with the pET22b vector expressing BOGUAY_2386 when induced with IPTG.
BL21 ∂DE3+ pET22B0651OGB	BL21 ∂DE3 strain with the pET22b vector expressing BOGUAY_0691 when induced with IPTG.

Supplementary Table 3. Genomic environment of candidate nitrite reductases, based on contig annotation in MacGregor et al. 2013a.

Contig No.	directionality	Gene annotation
**BOGUAY_0691 Orange Protein genomic region / upstream**		
0671	+	periplasmic nitrate reductase subunit NapA apoprotein
0672	+	periplasmic nitrate reductase subunit NapB
0684	+	hydrogenase small subunit
0685	+	Nitrate reductase gamma subunit (narG like)
0686	+	Fe-S oxidoreductase
0687	+	Fe-S oxidoreductase
0688	-	proton-translocating NADH-quinone oxidoreductase, chain N
0689	+	hypothetical
0690	+	hypothetical
**BOGUAY_0691 Orange Protein genomic region / downstream**		
0692	+	Hypothetical - acetyl xylan esterase
0692	-	Hypothetical - Haloacid hydrolase Superfamily protein
0693	-	NRAMP (natural resistance-associated macrophage protein) metal ion transporters
0694	+	Histidine kinase
0695	+	LytTR family transcriptional regulator
0696	+	Prolipoprotein diacylglyceryl transferase
**BOGUAY_2967 nirS protein genomic region / upstream**		
2963	+	Fic Fido domain (SUPERFAMILY SSF140931)
2964	-	hypothetical
2965	+	Mo-co oxidoreductase dimerisation domain-containing protein
2966	+	hypothetical protein
**BOGUAY_2967 nirS protein genomic region / downstream**		
2968	+	conserved domain protein
2969	+	Hypothetical protein
2970	+	Transposase DDE domain-containing protein / viral insertion ?
2971	+	RNA-directed DNA polymerase / viral insertion ?
2972	+	HNH endonuclease / viral insertion ?
2973	+	Group II catalytic intron / viral insertion ?
2974	+	conserved domain protein
**BOGUAY_2386 tetrathionate/nitrite reductase genomic region / upstream**		
2396	+	hypothetical protein
2395	+	putative redox protein
2394	+	tRNA-Met
2393	-	Putative restriction endonuclease
2392	-	dihydrofolate synthase / folylpolyglutamate synthase
2391	-	serine O-acetyltransferase
2390	+	sulfide-quinone oxidoreductase
2389	-	hypothetical protein
2388	-	undecaprenyl-diphosphatase
2387	+	hypothetical protein
**BOGUAY_2386 tetrathionate/nitrite reductase genomic region / downstream**		
2385	+	Thiosulfate reductase cytochrome b subunit
2384	+	chaperone TorD involved in molybdoenzyme TorA maturation
2383	+	thiosulfate reductase / polysulfide reductase chain A
2382	+	tetrathionate reductase subunit B
2381	+	formate dehydrogenase gamma subunit (EC 1.2.1.2)
2380	+	XisH protein (restriction endonuclease)
2379	+	XisI protein (restriction endonuclease)
